# The Origins of Enhanced and Retarded Crystallization in Nanocomposite Polymers

**DOI:** 10.3390/nano9101472

**Published:** 2019-10-16

**Authors:** Ahmad Jabbarzadeh

**Affiliations:** 1 Faculty of Engineering, School of Aerospace, Mechanical and Mechatronic Engineering, The University of Sydney, Sydney NSW 2006, Australia; ahmad.jabbarzadeh@sydney.edu.au; 2 Sydney Nano Institute, The University of Sydney, Sydney NSW 2006, Australia

**Keywords:** nanocomposites, cubic nanoparticles, polymer crystallization, molecular dynamics, hexacontane, Avrami constants, critical volume fraction, critical particle size

## Abstract

Controlling the crystallinity of hybrid polymeric systems has an important impact on their properties and is essential for developing novel functional materials. The crystallization of nanocomposite polymers with gold nanoparticles is shown to be determined by free space between nanoparticles. Results of large-scale molecular dynamics simulations reveal while crystallinity is affected by the nanoparticle size and its volume fraction, their combined effects can only be measured by interparticle free space and characteristic size of the crystals. When interparticle free space becomes smaller than the characteristic extended length of the polymer molecule, nanoparticles impede the crystallization because of the confinement effects. Based on the findings from this work, equations for critical particle size or volume fraction that lead to this confinement-induced retardation of crystallization are proposed. The findings based on these equations are demonstrated to agree with the results reported in experiments for nanocomposite systems. The results of simulations also explain the origin of a two-tier crystallization regime observed in some of the hybrid polymeric systems with planar surfaces where the crystallization is initially enhanced and then retarded by the presence of nanoparticles.

## 1. Introduction

Additives affect crystallization in natural systems and a large number of processes in polymers, pharmaceuticals, food, and chemical industries. Crystallization of polymers, in particular, is complex and is affected by a variety of factors that include temperature, cooling rate, and flow-induced deformation. When polymers crystallize in the presence of solid inclusions such as nanofillers, conducting nanoparticle agents, or nucleation agents, crystallization becomes even more complex. The amount of crystallinity in these hybrid systems has a significant impact on functional, mechanical, structural, and physical properties of the end product, and it is essential to control that by understanding the effect of additive nanoparticles. Despite many experiments to understand the effect of additives on crystallization, the results have often been contradictory. Therefore, control of crystallinity in hybrid molecular systems remains empirical at best. Experiments have shown that adding 6*wt%* 30 nm spherical silica nanoparticles to high-density polyethylene (HDPE) causes a 10% reduction in final crystallinity [1]. Experiments with polyethylene oxide (PEO) nanocomposites have shown similar results with a significant decrease in crystallinity by increasing *wt%* loading of ~25 nm size grafted nanosilica particles [2]. The slowdown in crystallization rate was also reported in more recent works for PEO/grafted silica nanocomposites [3]. Other experiments have attempted to determine the effect of talc nanoparticle (~30 nm in size) loading *wt%* in poly(ether ether ketone) (PEEK) nanocomposites crystallization [4] and have reported an optimal nanofiller %*wt* loading for maximum crystallinity. In contrast, adding 1–2*wt%* 500 nm zeolite to poly(butylene succinate) (PBS) [5] leads to approximately 1%–2% increased crystallinity. For polyethylene terephthalate (PET), adding Kevlar or glass nanofibers causes no measurable effect on crystallinity [6].

On the other hand, nylon/graphite nanocomposite systems [7] exhibit two-tier crystallization where initially the crystallization and nucleation is enhanced by additives; however, the final crystallinity and crystallization speed decrease. Such unusual crystallization behavior is also reported for poly(L-lactic acid) (PLLA)/nanoclay platelets with decreased crystallinity but accelerated growth [8]. Two-factor crystallization regimes are also reported for poly(ethylene oxide) PEO/Ti_3_C_2_T_x_ MXene nanocomposites [9] and nylon66/multiwall carbon nanotube nanocomposites [10].

The effect of nanoparticle size on the crystallization of poly(ethylene oxide) PEO/silica nanocomposites has been studied by Papananou et al. [11]. They have shown that for the same %v of nanoparticles, the amount of crystallinity decreases by decreasing the particle size, and that depending on the particle size there is a particular volume fraction where the crystallization will be impeded by the nanoparticles. Despite a plethora of experimental work, little is explored about the origins of two-tier crystallization and the effects of nanoparticle size and volume fraction. The field requires developing unifying universal equations to take into account the effect of volume fraction and particle size on confinement-induced effects on crystallization. The very fact that most experiments still report the additive nanoparticle content in weight% indicates unawareness on the impact of volume fraction, which will be shown here to be a critical parameter in crystallization. This issue becomes more critical when there is a big difference in the density of the added particles and the neat polymer melt. In that case, there is a significant disparity between weight% and volume%. As will be demonstrated here, volume fraction has a direct impact on the free space between the particles and the onset of confinement-induced crystallization for these systems.

Despite all challenges, there is considerable interest in controlling the crystallinity of polymers in the presence of additive nanoparticles by choosing the “right” size, shape and volume fraction. Therefore, it is imperative to unravel the complex effects of size and volume fraction of additives on the crystallization of nanocomposite polymer systems.

Discrete computational methods such as molecular dynamics simulations provide a valuable tool to explore the properties of nanomaterials. For nanocomposite materials, which are made by mixing polymer and nanoparticles, the effect of nanoparticles on the crystallization kinetics and final crystallinity have a profound impact on the final properties of the end product. Molecular dynamics simulations are capable to study the crystallization process and explore the kinetics of crystallization and structural effects under various conditions. As a method to calculate properties globally and locally at the nanoscale, molecular dynamics (MD) has been used by practitioners for several decades in the study of polymer crystallization [12,13]. We have used this method to study crystallization under quiescent and flow conditions and near surfaces [14,15,16]. Monte Carlo simulations of hard colloid particle crystallization near additive particles are done by Daan Frenkel’s group [17]. In their study, the nucleation of the system and the effect of additive particle size and curvature were explored.

In this paper, the results of MD simulations of a model polymeric system are presented, which includes clear evidence on the origin of the two-tier crystallization regime in hybrid and nanocomposite polymers. It is also revealed that the free interparticle space captures the combined effects of nanoparticle additive size and volume fraction on crystallization kinetics and final crystallinity. Such insight provides a roadmap to determine critical volume fraction or particle size of additives where a significant impact on final crystallinity and growth rate may be present because of the confinement effects posed by the additive nanoparticles. The findings have been used to explain the phenomena observed in relevant experiments.

## 2. Methodology

In this article, MD studies are presented for hexacontane (C_60_H_122_), a relatively long alkane that shows features of polymer crystallization such as folding. The system was studied as a pure polymer and also in a composite form mixed with cubic gold nanoparticles as additives. Previously, we have studied hexacontane in quiescent and flow-induced crystallization, and computational details can be found in references [14,15,16]. Depending on the size and volume fraction of the nanoparticles, a total of up to 2000 n-hexacontane molecules were simulated by a united atom model, with the intermolecular potential determined by Siepmann and co-workers [18,19], with a cut-off of ~1 nm for all potentials. The same cut-off was applied for polymer–polymer and particle–polymer interactions. The intramolecular interactions for the polymer included stretching, angle, and torsional potentials, and their parameters can be found in our earlier works [20,21]. The cubic gold nanoparticles with a face centred cubic FCC lattice size of 0.408 nm were cut from a slab to the closest desired size. A single, fixed additive nanoparticle was positioned at the center of the simulation box and was then filled with polymer molecules. Depending on the volume fraction and particle size, the initial simulation box size was 6.4–15.91 nm. For NPT (constant number of molecules, pressure, and temperature) simulation, the box size was variable and adjusted according to the pressure. Experimental evidence has shown the surface-to-surface interparticle distance during cooling of a nanocomposite polymer melt remains unchanged [2]. Therefore, fixing the particle position is a justifiable assumption. Periodic boundary conditions are applied in all three directions; therefore, the system represents a homogeneously distributed monodisperse, mono-orientational system of nanosized additives in a polymer matrix. All simulations were conducted under a fixed pressure (NPT) of 1 atm using the Nose–Hoover algorithm [22]. Temperature was kept constant by a Gaussian thermostat throughout the isothermal stages using the SLLOD algorithm [23]. The initial configuration of the pure and nanocomposite polymer melt was equilibrated at 500 K for 4.7 ns to create a fully amorphous system devoid of structural memory effects. The equilibration process for both the pure and nanocomposite C_60_H_122_ melt systems produced a polymer of approximately 771 kg/m^3^ density. This density was within 1.8% of the expected value (757 kg/m^3^) predicted by empirical equations given in reference [24]. The crystallization protocol started by cooling the system from 500 to 325 K at a cooling rate of 0.106 K/ps. The melting temperature of hexacontane is 368 K [25]; therefore, cooling to 325 K represents ~12% undercooking, which is necessary to speed up the crystallization. This non-isothermal stage lasted for 1.9 ns, followed by ~55 ns of isothermal–isobaric crystallization stage. All calculations were conducted using a simulation program developed by the author [26].

Hexacontane is a linear molecule and upon crystallization takes an all-trans configuration. Therefore, chord vectors connecting every other atom along the backbone attained parallel orientation relative to one another on the same molecule and with those in neighboring molecules that shared the same crystal lamella. To measure the degree of crystallinity, we detected the pairwise parallel orientation of chord vectors by using second- and fourth-rank correlation functions; $g_{2}(\Gamma )=\langle co2(\theta_{i}-\theta_{j})\rangle ;g_{4}(\Gamma )=\langle co4(\theta_{i}-\theta_{j})\rangle$, where *θ_i_* and *θ_i_* are the orientation angle of chord vectors *i* and *j*. These two functions are often used to detect nematic (parallel) and tetratic (herringbone) order [27]. For the chord vector, *i* position taken to be at the center of atom *i*, the pairwise order is calculated within a sphere of radius *r* = 0.5 nm, with atom *i* being at the center of this sphere. g_2_~g_4_~1 indicate 100% crystallinity and g_2_~g_4_~0 a completely amorphous system. Crystallinity can be measured as a global average or as a local parameter; g_2_ is used to represent the degree of crystallinity in results presented here. Further details can be found in references [14,15,16]. Kinetics of crystallization are studied by recording time-averaged crystallinity in time blocks of 75.1 ps.

## 3. Results and Discussion

### 3.1. The Effect of Particle Size

**Figure 1:**
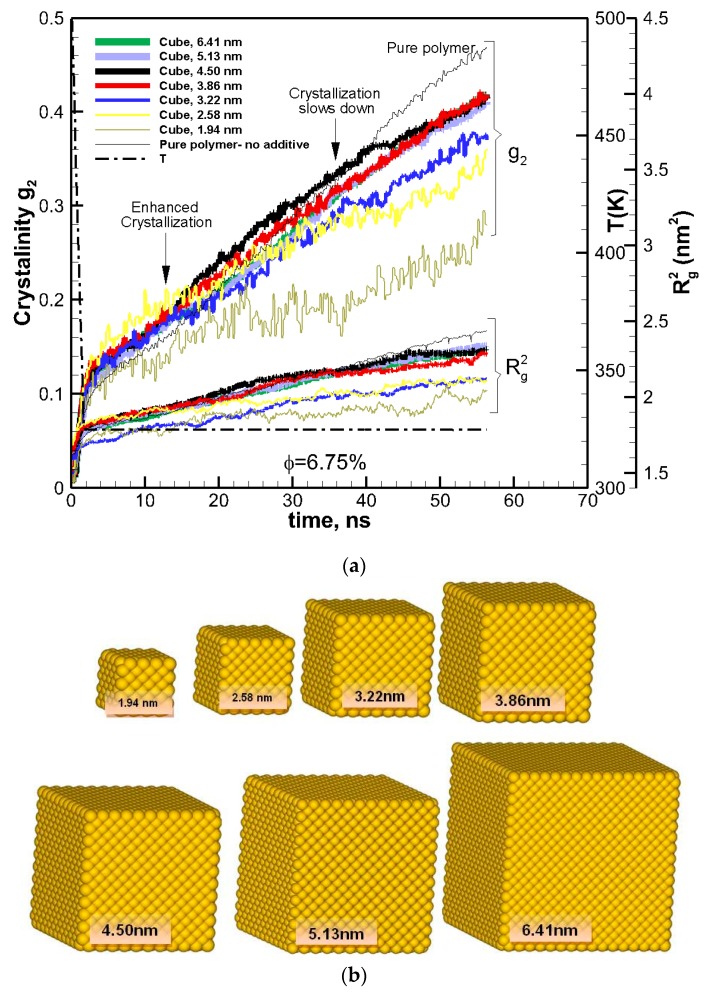
(**a**) Degree of crystallinity, g_2_, and square radius of gyration R^2^g shown as a function of time for pure polymer (hexacontane) (thin black solid lines) and nanocomposite polymer made of cubic gold nanoparticles of different sizes (solid lines of various thicknesses proportional to the size of additives). The volume fraction is approximately the same (~*φ* = 6.75%) for all composite polymer systems. Curly brackets group the corresponding curves for g_2_ and R^2^g. The arrows show two-tier crystallization regimes for nanocomposite polymers with cubic additives, where the crystallization is enhanced at the early stages and slowed down at final stages of crystallization. Temperature is shown by a dashed thick black line. (**b**) Cubic nanoparticles of various sizes used to create nanocomposite polymers.

To study the effects of the size of nanoparticles, the volume fraction was kept constant at *φ*~6.75%, and the cubic additive particle size varied from 1.94–6.41 nm. In Figure 1a, crystallization as a function of time is shown for the pure polymer as well for nanocomposite polymers with cubic nanoparticles of various sizes (shown in Figure 1b), all almost at the same volume fraction (~6.75%).

The results revealed a two-tier crystallization regime, where at the initial stages of crystallization the nanocomposite polymer systems showed enhanced crystallization in comparison to the pure polymer melt. However, as time proceeded, the rate of crystallizations slowed down for the nanocomposite systems, whereas it remained almost the same or increased for the pure polymer. After ~56 ns of crystallization, the amount of crystallinity for all nanocomposite polymers was lower than that for the pure polymer.

### 3.2. Effect of Nanoparticle Volume Fraction φ

**Figure 2:**
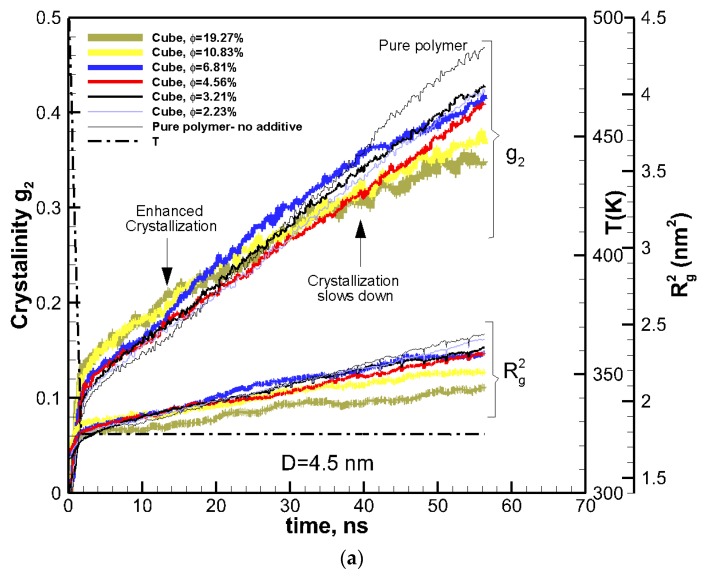

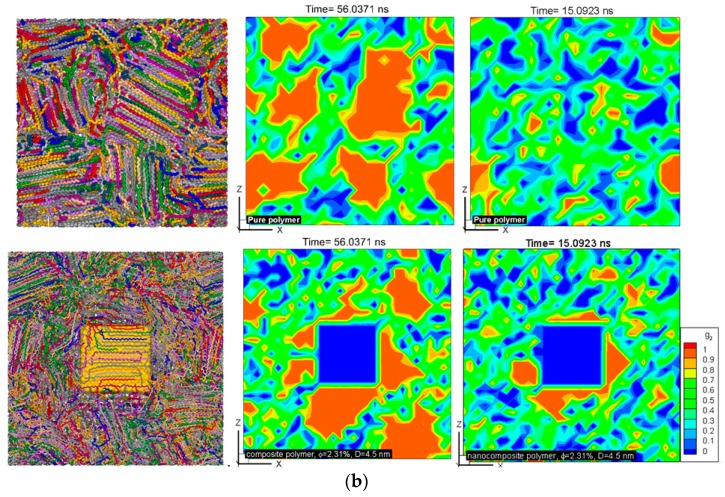
(**a**) The same as in Figure 1a, shown for nanocomposite systems of the same cubic additive particle size (*D* = 4.5 nm) and different volume fractions (solid lines of various thickness proportional to the volume fraction of additives). (**b**) Sliced contours of local crystallinity for pure (top row) and nanocomposite (bottom row, *D* = 4.5 nm, *φ* = 2.31%) polymers at the center of the simulation box are shown at ~15 ns (enhanced crystallization with nanoparticle additive) and ~56 ns (lower crystallization with nanoparticle additive). The red (color online) regions have more than 90% crystallinity (see also movies in the Supplementary Materials showing the slices across the simulation domain). The sliced snapshots show corresponding molecular orientations for pure and composite polymers at the end of crystallization (molecules are shown by different colors for clarity) (see also movies in the Supplementary Materials showing crystallization of molecules for the pure and nanocomposite polymers).

To study the effect of volume fraction, the size of additive particles was kept constant at ~4.5 nm, and the volume fraction varied between 2.33%–19.27%. The kinetics of crystallization are shown in Figure 2a for these nanocomposite polymers together with that of the pure polymer for comparison. The two-tier crystallization regime for nanocomposite systems was manifested again by enhanced crystallization at early stages and a subsequent slowdown at a later stage. The amount of crystallinity at 15 ns was higher in nanocomposite polymers than in the pure polymer. Furthermore, crystallinity increased with volume fraction. However, the effect was reversed towards the end of crystallization time at 56 ns, where all the nanocomposite polymers exhibited lower crystallinity than the pure polymer, and crystallinity decreased with increasing the volume fraction.

Contours of local crystallinity in Figure 2b showed that at ~15 ns, significant local crystallinity developed near the additive nanoparticles flat surfaces, whereas, for the pure polymer crystalline, domains were smaller and randomly distributed. These pictures present evidence that early enhanced crystallization is due to surface-induced crystallization by the additive nanoparticle. As such, this early stage of crystallization is most likely to be affected by the shape and surface area of an additive particle. The planar shape of the cubic particles here, therefore, contributes to this early enhancement of crystallization. The local crystallinity contours at ~56 ns (near the end of crystallization), showed (Figure 2b) that for the nanocomposite polymer, crystallization concentrated near the surface of the additive nanoparticle, whereas the pure polymer crystallized into randomly distributed and larger domains.

**Figure 3:**
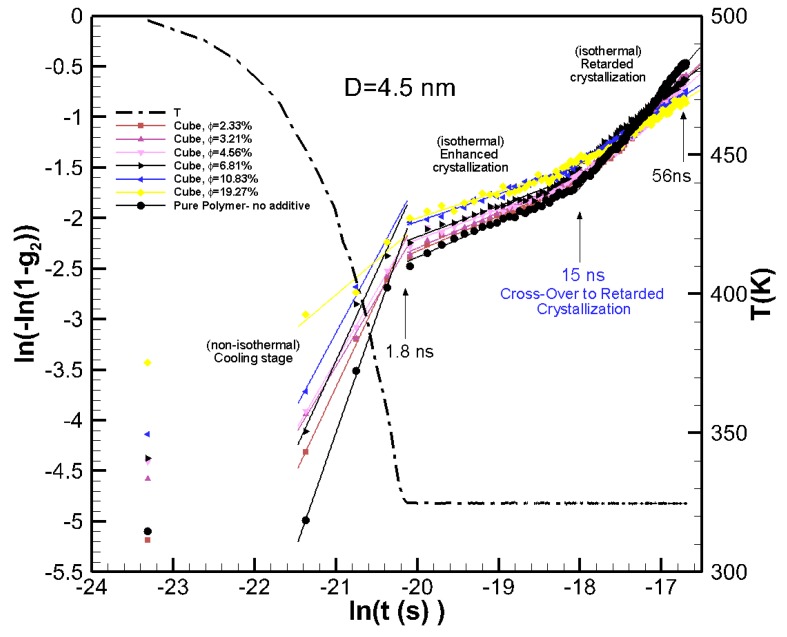
The Avrami plots for the crystallization kinetics data in Figure 2a for pure polymer and nanocomposite systems made from 4.5 nm particles at various volume fractions. The figure shows the growth rate and crossover from enhanced crystallization to retarded crystallization regimes.

To extract the growth rate and to identify the crossover from the enhanced crystallization regime to retarded crystallization regime, the results in Figure 2a are plotted in the Avrami scale. Avrami’s equation [28], $1-g_{2}=e^{-K(T)t^{n}}$, where *K(T)* is the growth rate function, and *n* is the Avrami’s exponent, which indicates dimensionality of the growth. By rearranging Avrami’s equation to $\ln[-\ln(1-g_{2})]=\ln K(T)+n\ln(t)$ we can extract ln*K*(*T*) and *n* by plotting ln(*–*ln(1*-g*_2_)) as a function of ln(*t*) and calculating the slope and intercept of the linear fit. Plotting in this logarithmic scale reveals new information. The results are plotted in Figure 3, which showed evident changes of the slope at the end of cooling (*t*~1.8 ns) and at *t*~15 ns, which seemed to be the crossover from enhanced crystallization to retarded crystallization regimes. The Avrami exponents and growth rate were extracted by a linear fit to the cooling, enhanced crystallization, and retarded crystallization regimes. The data are tabulated in Table 1. We see from the Avrami plots in Figure 3 that the initial crystallinity seeded by the particle was larger (larger values for ln(*–*ln(1*-g*_2_)) than those for the pure polymer. This initial crystallinity also increased with the volume fraction of the nanoparticles. At this cooling stage that lasted 1.8 ns, the pure polymer crystallized at a higher rate (see larger ln*K(T)* values for pure polymer in Table 1) than those seen for the nanocomposites. For the enhanced crystallization regime, which is under isothermal conditions, the nanocomposite systems had a higher crystallinity in comparison to the pure polymer. However, the growth rates for pure and nanocomposite systems were very close; the growth rate decreased slightly with increasing the volume fraction of the nanoparticles.

For the retarded crystallization regime, there was a significant difference in the growth rate of the pure polymer (ln*K(T)*~15.25) and nanocomposite polymers. For nanocomposite systems, the growth rate function ln*K(T)* reduced from ~12.9 to 7.36 as *φ* increases from 2.33% to 19.27%. We can see the crossover from enhanced crystallization to retarded crystallization started at about 15 ns. At this point there was a deflection in the growth rate for the pure polymer and nanocomposite systems, where pure polymer crystallized at much higher rate, whereas the nanocomposite systems showed much slower growth rates because of the emergence of confinement effects.

### 3.3. Combined Effects of Volume Fraction and Particle Size

**Figure 4:**
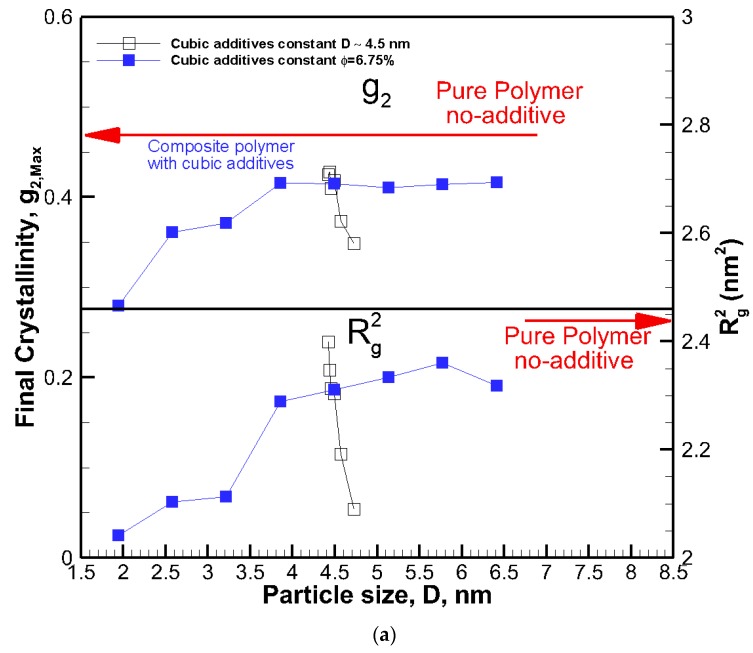

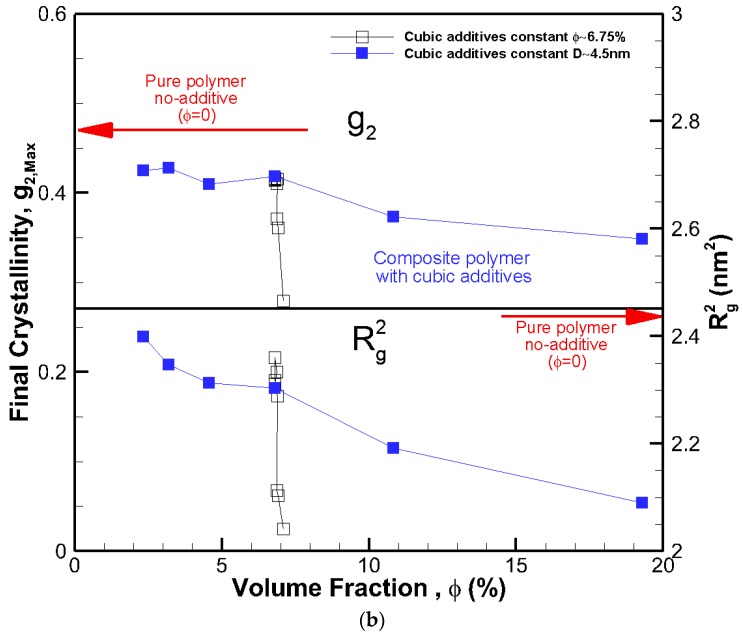
The final degree of crystallinity and square radius of gyration R^2^_g_ after 56.4 ns for all systems shown against cubic additive particle (**a**) size D, and (**b**) volume fraction *φ*. The red arrows show final crystallinity and R^2^_g_ for the pure polymer with no additive.

The final crystallinities for all systems against the size of the cubic addictive nanoparticles are shown in Figure 4a. The final crystallinity decreased as the particle size at the same volume fraction decreased. However, for roughly the same size particles (open symbols), we saw a significant impact on final crystallinity because of the change in volume fraction. This shows that the particle size alone is not a good indicator of the fate of crystallization. On the same note, the final crystallinity versus additive particle volume fraction, *φ*, is shown in Figure 4b, revealing a monotonic decrease in crystallinity as the volume fraction increased for the same particle size (filled symbols). However, at almost the same volume fraction ~6.75%, there was a significant change in final crystallinity because of the change in particle size (open symbols). Therefore, neither volume fraction nor particle size on their own was a suitable mean to control the final crystallinity or growth rate, and their combined effects must be considered.

To understand the origin of this two-tier crystallization regime and the impact of additive nanoparticle size and volume fraction, the ensemble-averaged square radius of gyration R^2^_g_ is also included in Figure 1a and Figure 2a. The final values of R^2^_g_ are shown in Figure 4a,b.

**Figure 5:**
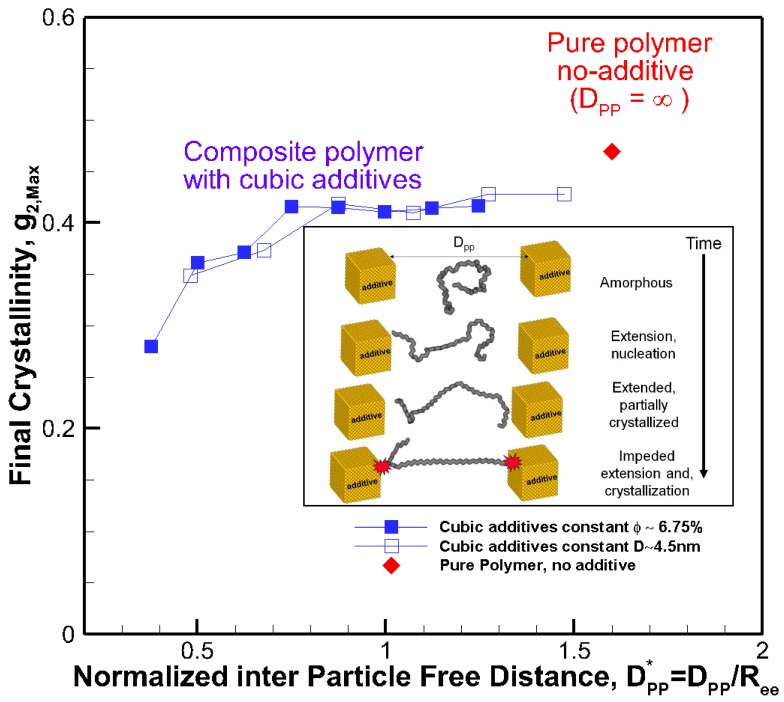
Final crystallinity is shown against the normalized interparticle free distance D*_pp_ = D_pp_/R_ee_ for nanocomposite polymeric systems with cubic nanoparticle additives of various sizes and volume fractions. The result for pure polymer without additives is shown with a single red rhomboid symbol for comparison. The inset shows a schematic of the molecular structure evolution from amorphous to partially extended, crystallized configuration and then impeded extension and crystallization due to the confinement effect caused by nanoparticle additives.

The results reveal that for all systems, initially, the radius of gyration increased as the time proceeded; however, for some nanocomposite systems, a plateau emerged indicating that molecular extension ceased or slowed significantly. It is well known and revealed in our earlier simulations and works by others [13] that molecular extension is necessary for polymer nucleation and subsequent crystallization. Such processes are responsible for flow-induced crystallization [14,15,29]. Therefore, the impediment caused by the presence of nanoparticle additives is the fundamental source of the slowdown in crystallization. For the amorphous hexacontane at *T* = 500 K, the calculated R^2^_g_ was 1.61 nm^2^. As the temperature dropped, the molecule extended, nucleated partially, and extended further as it crystallized; if the additive nanoparticle surface-to-surface distance is smaller than the size of the crystal to form between the nanoparticles, further extension and crystallization will be impeded or continue at a significantly reduced rate. The evidence of this reduced extension due to the presence of nanoparticle additives was manifested by a reduction in R^2^_g_ and can be seen in Figure 4a,b. Here R^2^_g_ reduced by increasing the volume fraction for the same particle size or by reducing the particle size for the same volume fraction. See also the inset of Figure 5. Final crystallinity is shown against the normalized interparticle free distance D*_pp_ = Dpp/R_ee_ for nanocomposite polymeric systems with cubic nanoparticle additives of various sizes and volume fractions. The result for pure polymer without additives is shown with a single red rhomboid symbol for comparison. The inset shows a schematic of the molecular structure evolution from amorphous to partially extended crystallized configuration and then impeded extension and crystallization due to the confinement effect caused by nanoparticle additives. See the inset in Figure 5 for a schematic representation of this phenomenon. Therefore, the slowdown of molecular extension with the additive nanoparticles is a confinement-induced phenomenon, whose severity depends on the available space between the nanoparticles.

### 3.4. Critical Volume Fraction and Particle Size

For an idealized, homogeneously distributed, monodisperse, mono-orientational cubic nanoparticles of volume fraction *φ* in a polymer matrix, one can show that the surface-to-surface interparticle free distance *D_pp_* is given by Equation (1):

(1) $$D_{pp}=D(\frac{1}{\sqrt[{3}]{\varphi }}-1),$$

where *D* is the size of cubic additive and *φ* is its volume fraction. This equation shows that for the same volume fraction, decreasing the particle size results in a decrease in interparticle free space. On a similar note, for the same particle size, increasing the volume fraction causes a decrease in *D_pp_*.

A fully extended hexacontane in an all-trans configuration has an end-to-end length of *R_ee_* = 7.62 nm and R^2^_g_ = 5.02 nm^2^. Therefore, R^2^_g_~2–2.4 nm^2^ observed at the end of the crystallization stage implied that most of the molecules were partially or once folded. A visual inspection of molecular conformation confirmed this (see snapshots in Figure 2b). Plotting the final crystallinity as a function of normalized D*_pp_ = *D_pp_/R_ee_*_,_ for all cases, regardless of particle size and volume fraction, in Figure 5 revealed a striking trend that explains the phenomenon observed about the effect of nanoparticle size and volume fraction. There is clear evidence that crystallinity increased by increasing the interparticle free distance. A plateau appeared as D*_pp_ approached ~0.75, that is *D_pp_*~5.7 nm, comparable to the size of an extended or partially folded chain. Beyond this point, no effect on final crystallinity was apparent. Therefore, D*_pp_ captured the combined effects of nanoparticle size and volume fraction on crystallization. D*_pp_ provides a measure to determine critical values of particle size for a given volume fraction or volume fraction for a given nanoparticle size, where the confinement effect becomes essential for a given nanocomposite polymeric system. Here, our polymer system was simple, and the characteristic polymer length was easy to establish. For more complex polymeric systems, one needs to establish the structural size, such as lamella thickness, spherulite, or other superstructures of interest for which the presence of nanoparticle additives will pose a growth impediment. Then, Equation (1) or alternative equations (for example, for spherical particles) $D_{pp}=D(\sqrt[{3}]{\frac{\pi }{6\phi }}-1)$ can be used to determine the critical particle size or volume fraction, where confinement effects become important. Using this approach and considering *R_c_* being the characteristic dimension representing the crystal structure size (molecule, lamella, etc.), we can obtain the following critical nanoparticle size (*D_cr_*) and volume fraction (*φ_cr_*) for cubical additives using Equation (1).

(2) $$D_{cr}=\frac{R_{c}\sqrt[{3}]{\varphi }}{1-\sqrt[{3}]{\varphi }}.$$

(3) $$\varphi_{cr}={\left(\frac{D}{R_{c}+D}\right)}^{3}.$$

These two parameters provide guidance where for *φ > φ_cr_* and *D* < *D_cr_* there will be significant confinement-induced slowdown effects on crystallization by the presence of cubic additives. It should be noted that the effects of free interparticle space presented here are independent of particle shape, and similar confinement-induced results are obtained by using spherical particles (not shown here), leading to alternative equations based on *D_pp_* for spherical particles.

## 4. Correlation with Experimental Results

Two modes of crystallization have been observed for poly(ethylene oxide) PEO and nanoplatelet Ti_3_C_2_T_x_ MXene nanocomposites [9]. They have observed that the crystallization rate increases for loading up to 0.33%v; however, at a critical loading somewhere between 0.33%v to 0.66%v, the rate of crystallizations slows down. In those experiments, exfoliated flat Ti_3_C_2_T_x_ nanoplatelets of *D* = 5 nm thickness were used. The thickness of PEO lamella is reported to be *R_c_* = 25 nm [2,30]. Using these values and Equation (3), one obtains *φ_cr_*~0.46%. This finding is in excellent agreement with the experimental observation of a critical volume fraction between 0.33%v to 0.66%v. Such relevance can also be found in considering experiments of work of Papananou et al. [11] who examined the PEO/silica nanocomposites of different particle sizes. Papananou et al. used uncoated spherical silica particles of 14, 37, and 134 nm diameters. They clearly showed a decrease in the crystallinity as the volume fraction of particles is increased. Papananou et al. reported that the crystallinity began to decrease at φ_cr_~30%v for nanoparticles of *D* = 134 nm. Using the critical volume fraction equation for spherical particles $\varphi_{cr-sphere}=\frac{\pi }{6}{\left(\frac{D}{R_{c}+D}\right)}^{3}$, one yields *φ_cr_*~31%v in excellent agreement. For the 37 nm particle, we yielded *φ_c_*_r_~11%v, which is also in agreement with Papananou et al. [11] experimental observations of ~12%v (Figure 5a in [11]). The prediction for the smallest particle (14 nm) was *φ_cr_*~2.4%. However, the experiments reported *φ_cr_*~20%, which may be related to the deviation of particles from spherical shapes [SI 11]. They also showed for the same volume fraction, decreasing the particle size resulted in a decrease in crystallinity, and critical volume fraction decreased by decreasing the particle size; this is also in agreement with that predicted in simulations here. Khan et al. [2] have shown the crystalline content of PEO and 25 nm grafted silica particle nanocomposites decreased, even at low loading of 10*wt%* (~8%v). The predicted critical volume fraction was *φ_cr_*~6.5%v*,* showing confinement-induced retardation of crystallization is expected at volume fractions of *φ* = 10–60 *wt%* (~8–46%v), which are used in Khan et al. [2] experiments. Khan et al. [2] reported reductions of about 5%–40% in relative crystallinity, confirming our predictions based on confinement-induced retardation of crystallization effects.

In summary, the results presented here establish that the origin of two-tier crystallization in nanocomposite polymer systems is a combination of surface-induced crystallization enhancement and confinement-induced retardation of crystallization. It was revealed that the combined effects of nanoparticle volume fraction and size on the crystallization of hybrid molecular systems could be captured by interparticle free distance, which is proportional to the particle size and inverse of the cubic root of its volume fraction. Furthermore, it is shown for interparticle free distances (and corresponding volume fraction/particle size) larger than characteristic crystal size, there is only a small impact on crystallization by additives, which relates to their surface, shape, and other effects.

## 5. Conclusions

In conclusion, it is shown by molecular dynamics simulations that the free distance between additive nanoparticles in nanocomposite systems determines the fate of crystallization, and equations for critical particle size and volume fraction are extracted for systems with cubical additives. Good agreements between experiments and those predicted based on this work could be demonstrated for critical volume fraction, the effect of particle size, and crystallization trends. These developed equations based on the findings here provide useful tools in formulating these systems for controlling the amount of crystallinity and its rate of growth for these hybrid molecular systems. They provide a quantitative measure of critical volume fraction for a given particle size and critical particle size for a given volume fraction where the crystallization is expected to be impeded by confinement-induced effects. The importance of using the volume fraction rather than percentage weight for additive particles is also highlighted by the findings in this study. While the results here are presented for a nanocomposite polymeric system, it is expected that these findings have a significant impact on the utilization of additive particles to control the crystallinity of a variety of hybrid polymeric and molecular systems. These processes will also have an impact on the morphology of the crystallized polymer, where additive nanoparticles can control the size of the crystals; however, it has not been explored in this work. The shape of the additive particles also may have an impact on the crystallization. Here, we considered cubic particles that showed evident two-tier crystallization. We are currently exploring the effect of particle shape and will publish the results soon. Such studies are necessary for obtaining a complete picture of the effect of nanoparticles on crystallization.

## Figures and Tables

**Table 1:** The Avrami growth function and exponent extracted from the linear fit to crystallization regimes shown in the Avrami plots in Figure 3.

Cubic Nanoparticles (*D* = 4.5 nm)						
Volume Fraction (%)	Cooling Stage		Enhanced Crystallization		Retarded Crystallization	
	ln*K(T)*(s^−*n*^)	*n*	ln*K(T)*(s^−*n*^)	*n*	ln*K(T)*(s^−*n*^)	*n*
2.33	33.34	1.72	4.56	0.35	12.90	0.81
3.21	24.90	1.35	4.70	0.35	12.29	0.77
4.56	25.68	1.39	3.71	0.30	11.35	0.72
6.81	33.72	1.77	3.34	0.31	10.17	0.65
10.83	28.29	1.49	3.45	0.27	7.98	0.52
19.27	11.52	0.68	3.27	0.26	7.36	0.50
0 (Pure Polymer)	41.4	2.17	4.49	0.344	15.25	0.94

## Supplementary Materials

The following are available online at https://zenodo.org/record/3463057#.XaeV5X9S9OQ, Movie 1.mov; Movie 2.mov; Movie 3.mov, Movie 4.mov.
